# Glucose-6-Phosphate Dehydrogenase Protects *Escherichia coli* from Tellurite-Mediated Oxidative Stress

**DOI:** 10.1371/journal.pone.0025573

**Published:** 2011-09-30

**Authors:** Juan M. Sandoval, Felipe A. Arenas, Claudio C. Vásquez

**Affiliations:** Departamento de Biología, Facultad de Química y Biología, Universidad de Santiago de Chile, Santiago, Chile; Laurentian University, Canada

## Abstract

The tellurium oxyanion tellurite induces oxidative stress in most microorganisms. In *Escherichia coli*, tellurite exposure results in high levels of oxidized proteins and membrane lipid peroxides, inactivation of oxidation-sensitive enzymes and reduced glutathione content. In this work, we show that tellurite-exposed *E. coli* exhibits transcriptional activation of the *zwf* gene, encoding glucose 6-phosphate dehydrogenase (G6PDH), which in turn results in augmented synthesis of reduced nicotinamide adenine dinucleotide phosphate (NADPH). Increased *zwf* transcription under tellurite stress results mainly from reactive oxygen species (ROS) generation and not from a depletion of cellular glutathione. In addition, the observed increase of G6PDH activity was paralleled by accumulation of glucose-6-phosphate (G6P), suggesting a metabolic flux shift toward the pentose phosphate shunt. Upon *zwf* overexpression, bacterial cells also show increased levels of antioxidant molecules (NADPH, GSH), better-protected oxidation-sensitive enzymes and decreased amounts of oxidized proteins and membrane lipids. These results suggest that by increasing NADPH content, G6PDH plays an important role in *E. coli* survival under tellurite stress.

## Introduction

The tellurium oxyanion, tellurite (TeO_3_^2−^), is especially harmful to prokaryotic cells mainly because of the generation of reactive oxygen species (ROS) [1]–[5]. In particular, tellurite-exposed *Escherichia coli* exhibits oxidative stress-sensitive [Fe-S] cluster-containing enzyme inactivation, increased protein carbonylation and lipid membrane oxidation, as well as activation of superoxide-responsive genes [3], [6]. In addition, tellurite causes thiol depletion, especially glutathione (GSH), that in turn causes oxidative stress [7], [8].

In response to superoxide-mediated stress, *E. coli* triggers a coordinated expression of a number of genes (*soxRS* regulon) whose biological role includes three different response levels: (i) prevention of oxidative damage, (ii) xenobiotic removal and recycling of damaged macromolecules and (iii) nicotinamide adenine dinucleotide phosphate (NADPH) regeneration [9]–[12]. In this context, intracellular NADPH levels are critical for maintaining a balanced redox status and therefore for survival [13].

Previous work from our laboratory has shown that NADPH metabolism is affected in cells exposed to the toxicant potassium tellurite. We observed that tellurite (Te^4+^) can be enzymatically reduced to elemental tellurium (Te^0^) by different microorganisms in a NAD(P)H-dependent manner [14]–[16]. Preliminary experiments have also indicated that the antioxidant response caused by tellurite-activated *soxRS* regulon might influence NADPH synthesis [3]. NADPH levels can be also affected because of non-enzymatic tellurite reduction by GSH or other intracellular reducing agents [7], [8].

Using a collection of mutants impaired in NADPH synthesis, we found that cells lacking glucose-6-phosphate dehydrogenase (G6PDH) were the most sensitive to tellurite. To a lesser extent, cells deficient in genes encoding isocitrate dehydrogenase (ICDH) or glutamate dehydrogenase (GDH) were also sensitive to the toxicant. Tellurite-exposed *E. coli* exhibited increased *zwf* expression which was paralleled by augmented G6PDH (protein amount and activity) and NADPH synthesis. Thus, upon *zwf* overexpression bacteria seems to be better protected against tellurite-induced stress.

## Results and Discussion

### Tellurite exposure results in augmented NADPH synthesis

Little is known about the *E. coli* antioxidant response when grown in the presence of tellurite. Previous reports by our group and others have shown increased superoxide dismutase activity in tellurite-exposed cells [3], [5]. In this context and since the metabolism of dinucleotides results altered in response to the oxidative stress-generating compounds gallium and menadione [17], [18] both NADP(H) and NAD(H) concentrations were determined to analyze whether tellurite exposure results in similar effects. Table 1 shows that while NADPH levels increased ∼30% NADH content was halved in tellurite-exposed wild type *E. coli*. Although decreased NADH concentrations may be counterproductive for energy generation [18], it may also represent a response to reduce the overall oxidative status of the cell. In fact, several NAD^+^-dependent enzymes such as α-ketoglutarate- and pyruvate-dehydrogenase complexes from prokaryotic or eukaryotic origins are selectively inhibited upon tellurite exposure ([16], [19], [20] Vásquez unpublished results). Surprisingly, the amount of oxidized dinucleotides (NAD^+^, NADP^+^) was not modified in the presence of the toxicant, a result that may be explained by NAD^+^ kinase activation in response to an oxidative stress-induced temporal NADPH depletion [18], [21], [22]. As expected, similar results were observed upon cell exposure to the superoxide-generating drug menadione (Table 1).

**Table 1:** Tellurite exposure results in augmented NADPH levels in *E. coli*.

	Treatment		
Cofactor	Control	K_2_TeO_3_ (2 µM)	Menadione (100 µM)
NADPH^a^	119.1±1.4	151.0±6.6*	155.2±9.1*
NADP^+^	46.9±7.8	47.7±5.0	44.4±6.9
NADH	53.0±3.1	27.0±4.9*	22.3±2.1*
NAD^+^	48.4±0.4	43.9±6.4	55.0±2.3

a NADP(H) (nmol mg prot^−1^) and NAD(H) (mmol mg prot^−1^) concentration in *E. coli* BW25113 extracts was determined spectrophotometrically at 340 nm as described in Methods. Values are the mean of 3 independent trials ± SD.

*P≤0.05 as compared to controls.

Aiming to identify genes whose products could participate in such a response, several strains lacking enzymes involved in NADPH synthesis were tested for tellurite sensitivity. While cells deficient in *gnd*, *maeB*, *pntA*, *pntB* or *udhA* genes were not affected by the toxicant, cells lacking ICDH (Δ*icdA*) or GDH (Δ*gdhA*) were ∼40–60% more sensitive when compared to the wild type strain. Interestingly, cells devoid of G6PDH activity (Δ*zwf*) exhibited ∼2-fold more sensitivity to tellurite, H_2_O_2_ and diamide (Fig. S1). In addition, ICDH, GDH and G6PDH activity increased ∼30, 50 and 60% when wild type cells were exposed to tellurite (Table 2), suggesting that these activities -mainly G6PDH- are most probably involved in increasing NADPH levels to face tellurite stress. This last assumption was confirmed by determining ICDH and GDH activity in extracts from tellurite-exposed Δ*zwf* cells: while ICDH activity decreased by ∼25% regarding the untreated control, GDH activity results almost undetectable (Table 2). In the absence of tellurite, the Δ*zwf* strain showed decreased (∼20%) NADPH levels regarding the wild type control and tellurite exposure did not modify them significantly (not shown).

**Table 2:** Tellurite induces NADPH-dependent enzymatic activities in *E. coli*.

	BW25113		Δ*zwf*	
Enzyme	Control	K_2_TeO_3_	Control	K_2_TeO_3_
ICDH	0.095±0.01	0.121±0.01*	0.011±0.01	0.084±0.04*
GDH	7.7±0.7	11.8±1.3*	0.3±0.07	0.6±0.1*
G6PDH	7.0±1.4	11.3±1.7*	ND	ND

Enzymatic activity (µmol NADPH min^−1^ mg prot^−1^) was determined spectrophotometrically at 340 nm as described in Methods. Values are the mean of 4 independent trials ± SD.

*P≤0.05 as compared to controls. ND, not detected.

### ROS generation and not thiol depletion is the primary signal for tellurite-induced *zwf* expression

**Figure 1:**
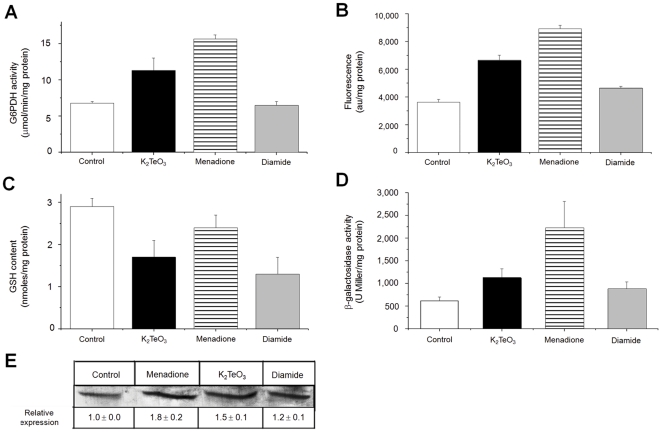
Tellurite induces G6PDH because of ROS formation and not thiol depletion in *E. coli*. (A) G6PDH activity was determined spectrophotometrically at 340 nm as described [49]. (B) Intracellular ROS levels were analyzed using the oxidation-sensitive probe H_2_DCFDA (2′,7′-dichlorofluorescein diacetate) using an Applied Biosystems equipment CytoFluor 4000 Fluorescence Multi-well Plate Reader (excitation 490 nm, emission 519 nm) and normalized to protein concentration. (C) GSH content was assessed as described previously [56] with modifications. (D) β-galactosidase activity was determined in extracts of the reporter *E. coli* GC4468 *zwf*::*lacZ* strain [42] by monitoring the hydrolysis of *o*-nitrophenyl-β-D-galactopyranoside as described [43]. (E) Western blotting of G6PDH was analyzed using a specific *in-house* made antiserum. Band intensities were analyzed using the Gel-Pro Analyzer Program software, version 3.1. Relative expression was referred to that of control cells. The strain used in A–C and E was *E. coli* BW25113. *E. coli* cells were left untreated (control, white) or treated with 2 µM tellurite (black), 100 µM menadione (horizontal stripes) or 500 µM diamide (grey) for 30 min. Values are the mean ± SD of 3–4 independent trials. au, arbitrary units.

To determine if tellurite-mediated ROS generation [3] or thiol depletion [7], [8] is responsible of G6PDH activation, the effect of the superoxide-generating compound menadione [23] or the thiol-specific reagent diamide [24] was determined. Clearly G6PDH activity was induced by tellurite and menadione but not by diamide (Fig. 1A); this was true also for ROS production (Fig. 1B). In spite that tellurite-exposed cells exhibited a decreased GSH content (∼50%), this effect does not seem to be the primary signal inducing G6PDH activity (Figs. 1C). To assess if the observed tellurite-mediated increase of G6PDH activity was related to *zwf* induction, β-galactosidase activity was determined in the *E. coli* reporter strain *zwf*::*lacZ*. Fig. 1D shows that β-galactosidase activity increased 2- and 4-fold in extracts from tellurite- or menadione-exposed cells, respectively, when compared to untreated controls. No augment of β-galactosidase activity was observed in diamide-exposed cells (Fig. 1D). Increased G6PDH activity was paralleled by an augmented G6PDH protein, as shown by Western blotting (Fig. 1E). These results are in agreement with the *soxRS* regulon induction occurring at the onset of oxidative stress [25]. No induction of G6PDH activity was observed in tellurite-exposed Δ*soxRS E. coli* (not shown), again indicating that the underlying signal for G6PDH induction is related to tellurite-induced ROS generation.

### Tellurite treatment induces G6P accumulation in *E. coli*

Several lines of evidence suggest that the metabolic adaptation model of Singh *et al.* [18] is associated with flux changes in central metabolic pathways. In this context, cell exposure to oxidants results in altered NADH/NADPH content [18], *soxRS*-mediated *zwf* activation [25] and a shifting of glucose catabolic flux from glycolysis to the pentose phosphate pathway (PPP) [26]–[28]. To determine if a similar event could explain the observed tellurite-mediated increase of NADPH levels (Table 1), the intracellular concentration of glucose-6-phosphate (G6P) was assessed. The G6P content increased ∼50% in tellurite-exposed cells as compared to untreated controls (Table 3). As expected, the activity of the G6P suppliers PtsG (glucose-specific transporter of the phosphotransferase system) and Pgi (phosphoglucose isomerase), involved in a *soxRS*-controlled antioxidant mechanism [28], increased ∼2–3 fold under tellurite stress (Table 3). Preliminary results from our laboratory also indicate that while augmented *pgi* transcription occurs upon tellurite exposure, the activity of the enzymatic regulators phosphofructokinase and pyruvate kinase is significantly decreased, suggesting that the glycolytic pathway is down-regulated in these conditions (Vásquez, unpublished data).

**Table 3:** Tellurite exposure induces G6P accumulation in *E. coli*.

	Treatment		
	Control	K_2_TeO_3_	Menadione
G6P^a^	6.1±0.5	9.2±2.3*	5.4±0.2
PtsG^b^	11.9±3.3	29.6±7.9*	28.1±9.1*
Pgi^b^	0.26±0.1	0.56±0.1*	0.46±0.1*

a Intracellular G6P concentration (nmol mg prot^−1^);

b Enzymatic activity (µmol NADPH min^−1^ mg prot^−1^) was determined spectrophotometrically at 340 nm as described in Methods. Values are the mean of 3 independent trials ± SD.

*P≤0.05 as compared to controls.

### *zwf* expression is involved in the *E. coli* response to tellurite stress

**Figure 2:**
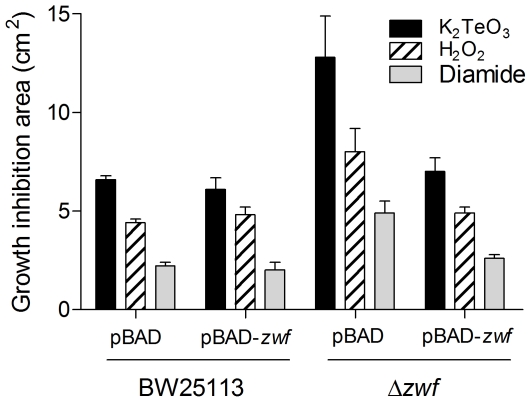
Effect of *zwf* expression on the *E. coli* sensitivity to oxidative stress elicitors. Growth inhibition zones were determined for wild type (BW25113 pBAD), *zwf*-overexpressing (pBAD-*zwf*), mutant (BW25113 Δ*zwf*::*kan*) and genetically complemented (BW25113 Δ*zwf*::*kan* pBAD-*zwf*) strains as described [41]. Briefly, cells were grown to OD_600_∼0.5, diluted and spread on LB plates. After air drying, ten microliters of tellurite (40 mM), H_2_O_2_ (10 M) or diamide (100 mM) were deposited on sterile disks in the centers of the plates. Results were determined after 24 h. Values are the mean of 4–5 independent trials ± SD.

To further unveil the role of G6PDH in the cellular response to tellurite, the effect of overexpressing or eliminating the *E. coli zwf* gene was carried out (see Table S1 for strain genotypes). Curiously, cells overexpressing *zwf* did not show increased tolerance to tellurite and resistance levels similar to those exhibited by wild type controls were observed in genetically-complemented strains (Fig. 2). Similar results were obtained when hydrogen peroxide or diamide were used. Since the absence of *zwf* results in increased sensitivity to tellurite and other stress-generating compounds (Figs. 2 and S1), it was expected that inducing *zwf* expression would reverse this effect, which was the case in *zwf*-complemented cells (Fig. 2). Overexpressing *zwf* did not generate increased resistance to these toxicants in either LB (Fig. 2) or M9-minimal medium (not shown). Similar results have been observed in *Salmonella enterica* serovar Typhimurium and *E. coli* exposed to H_2_O_2_, S-nitroso-glutathione or paraquat [29], [30].

In agreement with results of Table 1, tellurite-treated *E. coli* carrying pBAD vector alone also showed increased (∼30%) NADPH synthesis regarding the respective controls (Table 4). In pBAD-carrying Δ*zwf* cells NADPH levels decreased ∼20% while those of NADP^+^ increased ∼50%, an effect that was unchanged in the presence of the toxicants (Table 4). In turn, upon *zwf* overexpression NADPH levels increased ∼30% in the absence of toxicants while genetically-complemented Δ*zwf* cells exhibited dinucleotide levels similar to those of the wild type strain (Table 4). These results suggest that the protective effect of G6PDH activity (or its product NADPH) occurs during the *soxRS*-mediated response in cells facing stress [22] and that any further increase of activity is not reflected in higher tellurite resistance. Restitution of the resistance phenotype in the complemented strain (Fig. 2 and Table 4), therefore seems to result from increased NADPH levels.

**Table 4:** Effect of *zwf* expression and toxicant exposure on NADPH concentration.

Strain	Plasmid	Treatment	NADP^+a^	NADPH^a^	[NADP^+^+NADPH]
BW25113	pBAD	Control	46.9±7.8	119.1±7.7	166.0
		K_2_TeO_3_	47.7±5.0	157.0±6.6*	204.8
		Menadione	44.4±6.9	155.2±9.1*	199.6
BW25113	pBAD-*zwf*	Control	35.0±3.5	156.8±13.5	191.8
		K_2_TeO_3_	47.1±1.9*	218.3±6.6*	265.4
		Menadione	42.6±4.6	229.6±10.0*	272.2
Δ*zwf*	pBAD	Control	68.7±3.2	100.4±2.8	169.1
		K_2_TeO_3_	70.2±8.2	124.4±10.0	194.6
		Menadione	73.8±6.8	98.1±8.7	172.4
Δ*zwf*	pBAD-*zwf*	Control	45.5±4.1	138.8±3.0	184.3
		K_2_TeO_3_	57.9±3.1	156.0±8.5*	214.1
		Menadione	46.8±8.3	141.9±4.0	188.7

a nmol/mg protein. Values are the mean of 3 independent trials ± SD.

* , P≤0.05 as compared to control.

**Figure 3:**
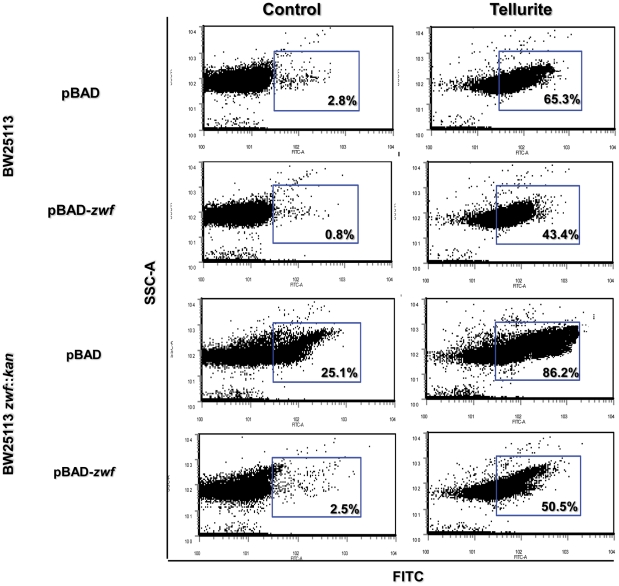
Effect of *zwf* expression on ROS content. ROS content was assessed in the indicated strains by flow cytometry using the oxidation-sensitive probe H_2_DCFDA. Expression of *zwf* was induced in the presence of L-arabinose (0.2%). Cells were incubated for 30 min in the absence or in the presence of 2 µM tellurite, washed and incubated with 2 mM H_2_DCFDA for 30 min in the dark, washed again and diluted 1∶10 with PBS buffer. Fluorescence intensity was determined using a Becton Dickinson model FacsCanto II equipment equipped with an argon laser (excitation 490 nm, emission 519 nm) [45]. The per cent of cell population that was positive for fluorescence is indicated (blue rectangles). A representative dot plot of 3 independent trials is shown. FITC, fluorescence intensity; SSC-A, cell complexity.

Next, the effect of *zwf* expression on several markers of tellurite-triggered oxidative stress was investigated. All tested strains showed increased ROS content in the presence of tellurite as compared to controls (Fig. 3). While Δ*zwf* cells showed a significant ROS increase (∼20%) even in the absence of the toxicant, the genetically-complemented strain showed ROS levels comparable to unexposed wild type controls. Also, *zwf* overexpression reduced tellurite-induced ROS content by ∼20–30% in BW25113 pBAD-*zwf* and Δ*zwf* pBAD-*zwf* cells. These results suggest a direct relationship between *zwf* expression (resulting in NADPH generation) and intracellular ROS content (Fig. 3). A putative explanation for these findings may lay in NADPH acting -apart from its role in maintaining the cellular redox state- as scavenger of various radical species [31], [32].

Since tellurite-mediated ROS generation causes oxidative damage in several macromolecules (protein carbonylation, membrane lipid peroxidation) [3], we tested whether *zwf* expression had a potential effect on these oxidation markers (Fig. S2). In the presence of the toxicant, both classes of damage were induced in Δ*zwf* cells, probably as consequence of diminished antioxidant ability (lower NADPH content) or the high basal ROS content displayed by these cells (Table 4 and Fig. 3). In *zwf*-expressing mutants, carbonylated proteins and lipid peroxides levels were restored to those observed in wild type controls (Fig. S2). Evidence about the role of G6PDH and/or NADPH in regulating the oxidation status of protein and lipid macromolecules in bacterial systems is scarce. However, it has been reported that *Saccharomyces cerevisiae* lacking Δ*idp2* and Δ*zwf1*, encoding the cytoplasmic isoforms of ICDH and G6PDH, respectively, displays increased levels of membrane protein oxidation [33], [34].

On the other hand, it has been previously shown that cell exposure to tellurite also affects essential [Fe-S] cluster-containing enzymes as aconitase and fumarase [6] and redox equivalents from G6PDH-synthesized NADPH can be first transferred to NADPH-dependent ferrodoxin/flavodoxin reductase and then to oxidatively-damaged [Fe-S] clusters [30]. In this context, it was found that *zwf* overexpression results in augmented fumarase activity, even in the presence of toxicants (Fig. S3). Increased NADPH levels (in *zwf*-overexpressing cells, Table 4) could probably protect and/or repair more efficiently [Fe-S] cluster-containing enzymes during the *soxRS* response [22], [30].

Finally, since tellurite also triggers GSH oxidation resulting in important changes in the cell's redox status [7], [8], [35], it was assessed if *zwf* expression influences intracellular GSH levels. As expected, the GSH content decreased (∼45%) in the presence of tellurite and diamide, suggesting that *zwf* expression and thus NADPH (Table 4), could participate in regulating GSH levels (not shown). Preliminary results indicate that the observed increase in GSH levels is not related to glutathione reductase activity (not shown). Experiments aiming to determine which route is being used to recover GSH levels in tellurite-exposed *E. coli* [35] are under way in our laboratory.

## Materials and Methods

### Bacterial strains and plasmids

*E. coli* strains and plasmids used in this study are listed in Table S1. *E. coli* BW25113 chromosomal DNA and the specific primers indicated was used to amplify the *zwf* gene. The PCR product was ligated to pBAD TOPO (Invitrogen) vector resulting in plasmid pBAD-*zwf*. Insert orientation was confirmed by *Sal*I digestion and PCR. Plasmids pBAD and pBAD-*zwf* were transformed into *E. coli* BW25113 and Δ*zwf* strains.

### Growth conditions and toxicant treatment

Bacteria were routinely grown in LB medium [36] at 37°C with vigorous shaking to OD_600_∼0.5. When required, ampicillin (100 µg ml^−1^) or kanamycin (50 µg ml^−1^) was added to the medium. Unless otherwise stated, compounds tested were used at final concentrations of 2.0 µM (tellurite), 100 µM (menadione), 1 mM (H_2_O_2_) and 500 µM (diamide). Gene induction was carried out in the presence of 0.2% L-arabinose.

### Determination of growth inhibition zones

Growth inhibition zones were determined as described previously [37]. Briefly, cells were grown to OD_600_∼0.5, diluted and spread on LB plates (2%). After air drying, toxicants to be tested were deposited on sterile filter disks previously placed at the centers of the plates. Plates were incubated overnight at 37°C.

### Enzyme purification

*E. coli* BL21(DE3) harboring plasmid pET17-G6PDH [30] was used to purify G6PDH. Cells were grown to OD_600_∼0.5 and induced with 1 mM IPTG for 5 h with vigorous agitation. After disrupting by sonication, crude extracts were prepared in 20 mM sodium phosphate buffer, pH 7.4, that contained 0.5 M NaCl and 20 mM imidazole. Proteins were purified by HisTrap HP (Amersham) affinity column chromatography as recommended by the vendor.

### Transcriptional analysis

Overnight cultures of *E. coli* GC4468 carrying a chromosomal *zwf*::*lacZ* fusion [38] were diluted 1∶1000 with fresh LB medium and grown at 37°C to OD_600_∼0.2. Samples (in triplicate) were removed to assay for β-galactosidase by monitoring the hydrolysis of *o*-nitrophenyl-β-D-galactopyranoside as described [39].

### Western blotting

*E. coli* cultures (10 ml) were centrifuged and suspended in 0.5 ml of 50 mM phosphate buffer, pH 7.4, which contained 0.1 mM phenylmethylsulfonyl fluoride. Cells were disrupted by sonication and G6PDH content was analyzed by SDS-PAGE and immunoblotting using a specific antiserum. Band intensity was analyzed using the Gel-Pro Analyzer Program software, version 3.1.

### Detection of reactive oxygen species (ROS)

To determine intracellular ROS, the oxidation-sensitive probe H_2_DCFDA (2′,7′-dichlorofluorescein diacetate, Calbiochem) was used. Aerobically grown cells in LB medium (OD_600_∼0.5) were split up into 4 identical aliquots and treated individually for 30 min with the different compounds tested. Cultures (1 ml) were sedimented and cells washed with potassium phosphate buffer 10 mM, pH 7.0, and incubated for 30 min with an equal volume of buffer containing 20 µM H_2_DCFDA (in dimethylsulfoxide) in the dark. After washing, cells were disrupted by sonication and extracts (100 µl) were loaded in triplicate in 96-well plates. Fluorescence intensity was determined using an Applied Biosystems equipment CytoFluor 4000 Fluorescence Multi-well Plate Reader (excitation 490 nm, emission 519 nm) and normalized to protein concentration as described earlier [3], [40].

Assessing intracellular ROS by flow cytometry was performed in the same way with minor modifications. Tert-butylhydroperoxide (100 µM) was used as positive control (not shown). Cells were incubated with 2 mM H_2_DCFDA for 30 min in the dark, washed and diluted 1∶10 with PBS buffer (137 mM NaCl, 2.7 mM KCl, 10 mM Na_2_HPO_4_, 2 mM KH_2_PO_4_, pH 7.4) [41]. Fluorescence intensity was determined using a Becton Dickinson model FacsCanto II equipment equipped with an argon laser (excitation 490 nm, emission 519 nm).

### Determination of protein carbonyl group content

Protein carbonyl group content was determined as previously described [42], [43]. Nucleic acid-free *E. coli* extracts were mixed with 4 volumes of 10 mM dinitrophenylhydrazine (dissolved in 2 M HCl) and incubated for 1 h at room temperature. Proteins were precipitated with 1 volume of cold 20% trichloroacetic acid and centrifuged at 10,000*g* for 10 min. After washing 3 times with ethanol∶ethyl acetate (1∶1), the pellet was dissolved with 450 µl of 50 mM dithiothreitol in 6 M guanidine-HCl. Carbonyl content was determined at 370 nm using a molar absorption coefficient of 22,000 M^−1^ cm^−1^ [42].

### Determination of membrane lipid peroxides

Membrane lipid peroxides were determined as described previously [44]. Toxicant-treated *E. coli* was centrifuged and suspended in 0.5 ml of a solution that contained 50 mM Tris-HCl, pH 7.4, and 1% SDS. After sonication samples were washed with water and dried pellets were dissolved in methanol∶chloroform (2∶1) and kept at room temperature with agitation for 1 h. Samples were then treated with 300 µl of the FOX II reactant (0.1 mM xylenol orange, 0.25 mM ammonium ferrous sulfate, 25 mM H_2_SO_4_, 4 mM butylated hydroxytoluene, in 90% methanol), mixed and let to stand at room temperature for 1 h. Membrane lipid peroxide content was determined at 560 nm using a molar absorption coefficient of 45,200 M^−1^ cm^−1^ [44].

### Determination of enzyme activity

Cells from 10 ml cultures were disrupted by sonication and extracts cleared by centrifugation. Aliquots of cell-free extracts were assayed for glucose-6-phosphate dehydrogenase [45], isocitrate dehydrogenase [46], NADP^+^-glutamate dehydrogenase [47], fumarase [48] and glutathione reductase [49]. Protein concentration was determined as described by Bradford using bovine serum albumin as standard [50].

### Determination of dinucleotide concentration

Duplicated samples were used for the selective extraction of dinucleotides as described earlier [51]. Briefly, cells were centrifuged at 13,000*g* for 2 min and immediately frozen in a dry ice-ethanol bath. Samples were treated with 250 µl of 0.2 M HCl or 0.2 M NaOH for extracting NAD(P)^+^ or NAD(P)H, respectively. Dinucleotides were extracted after incubating for 10 min at 100°C and centrifuging at 5,000*g* for 5 min to remove the cell debris. Supernatants were transferred to fresh tubes and kept on ice until use. Both NADP^+^ and NADPH were assessed spectrophotometrically using commercially available G6PDH and glutathione reductase, respectively [52]. NADP^+^ and NADPH standards from 0.01–1.0 mM were used to calibrate the assays.

Intracellular concentrations of NAD^+^ and NADH were assessed spectrophotometrically using NADH-dependent alcohol dehydrogenase as described previously [53] with modifications [54]. Standards of NAD^+^ and NADH from 0.05–0.75 mM were used to construct a calibration curve.

### Determination of GSH content

After tellurite or diamide treatment, cells were washed twice with ice-cold phosphate-buffered saline and centrifuged at 4°C for 2 min at 10,000*g*. Pellets were suspended in 100 µl of 5-sulfosalicylic acid (SSA) (5%, w/v), frozen in liquid nitrogen, thawed twice, centrifuged at 4°C and kept at −80°C until use. Total glutathione (GSH+GSSG) was determined as described previously [55]. Reduced GSH was calculated from total glutathione to which oxidized glutathione (GSSG) was subtracted. GSSG was determined using 2-vinylpyridine (M2VP) as described earlier [56] with minor modifications. Both GSH and GSSG standards from 0 to 0.5 mM were used to calibrate the assay.

### Determination of G6P concentration

Cultures (1 ml) -in duplicate- were centrifuged at 13,000*g* at 4°C for 2 min, washed and sonicated. Cell lysates were cleared by centrifugation at 13,000*g* for 10 min at 4°C. Extracts were boiled for 10 min, chilled and centrifuged at 13,000*g* for 10 min at 4°C. Supernatants were used immediately. Samples (50–200 µl) were incubated in a reaction buffer that contained 50 mM Tris-HCl, pH 7.4, 10 mM MgCl_2_, 0.7 mM NADP^+^ and 0.5 U ml^−1^ G6PDH. G6P concentration was assessed spectrophotometrically at 340 nm as described [57]. Standards of G6P from 0.005 to 0.25 mM were used to calibrate the assay.

### Data analysis

In general, results were expressed as the mean ± the standard deviation. Differences between experimental groups were analyzed using one-way ANOVA. P values less than 0.05 were considered statistically significant.

## Supporting Information

Figure S1 **Sensitivity of various** ***E. coli*** **strains impaired in NADPH synthesis to oxidative stress elicitors.** Growth inhibition zones (cm^2^) were determined for wild type and several strains deficient in NADPH synthesis essentially as described in Fig. 2. Results were determined after 24 h. Values are the mean of 4–5 independent trials ± SD. BW25113 (wild type), Δ*zwf* (glucose-6-phosphate dehydrogenase), Δ*gnd* (6-phosphogluconate dehydrogenase), Δ*icdA* (isocitrate dehydrogenase), Δ*maeB* (NADP^+^-dependent malic enzyme), Δ*gdhA* (glutamate dehydrogenase), Δ*pntA* (pyridine nucleotide transhydrogenase, α-subunit), Δ*pntB* (pyridine nucleotide transhydrogenase, β-subunit), Δ*udhA* (soluble pyridine nucleotide transhydrogenase).
(TIF)

Figure S2 **Effect of** ***zwf*** **expression on macromolecule oxidation.** Oxidized cytoplasmatic proteins (A) and total membrane lipid peroxides (B) were assessed in the indicated strains. Cells were grown in LB-arabinose in the absence of toxicant (white bars) or exposed to 2 µM tellurite (black bars) or 100 µM H_2_O_2_ (stripes) for 30 min. Values are the average of 3 independent trials ± SD.
(TIF)

Figure S3 **Effect of** ***zwf*** **expression on fumarase activity.** Total fumarase activity was assessed as described in Methods. The indicated strains were grown in LB-arabinose in the absence of toxicants (white bars) or exposed for 30 min to 2 µM tellurite (black bars) or 100 µM menadione (stripes) for 30 min. Values are the average of 3 independent trials ± SD.
(TIF)

Table S1 ***E. coli*** **strains, plasmids and primers used in this study.**
(DOCX)
