# Supplementary figures and images for: Glucose-6-Phosphate Dehydrogenase Protects Escherichia coli from Tellurite-Mediated Oxidative Stress
Source: PLoS One. 2011 Sep 30;6(9):e25573. doi: 10.1371/journal.pone.0025573 (PMC3184162; doi:10.1371/journal.pone.0025573)

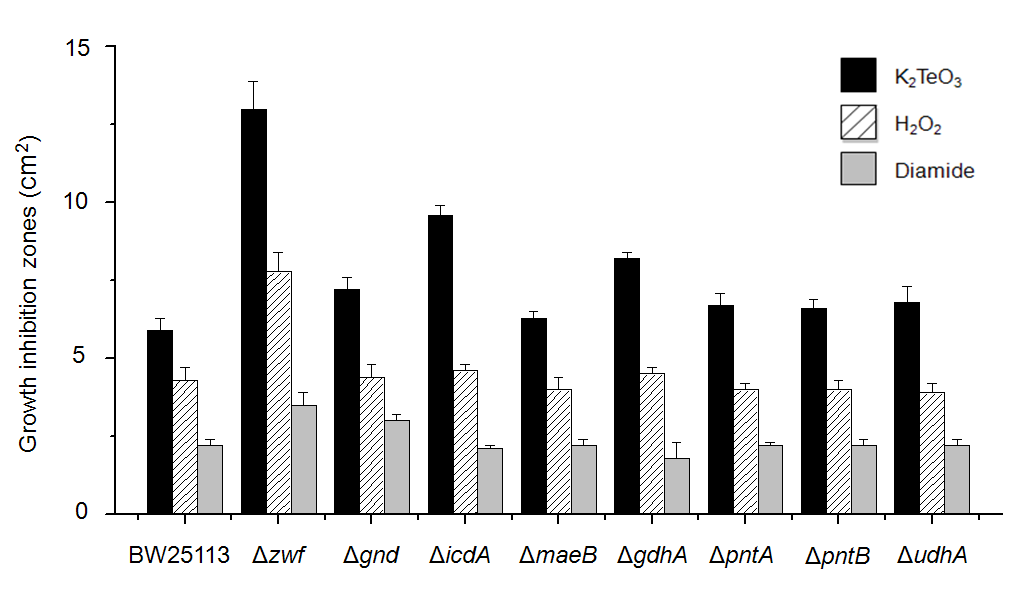

Supplement: Figure S1 — Sensitivity of various E. coli strains impaired in NADPH synthesis to oxidative stress elicitors. Growth inhibition zones (cm2) were determined for wild type and several strains deficient in NADPH synthesis essentially as described in Fig. 2. Results were determined after 24 h. Values are the mean of 4–5 independent trials ± SD. BW25113 (wild type), Δzwf (glucose-6-phosphate dehydrogenase), Δgnd (6-phosphogluconate dehydrogenase), ΔicdA (isocitrate dehydrogenase), ΔmaeB (NADP+-dependent malic enzyme), ΔgdhA (glutamate dehydrogenase), ΔpntA (pyridine nucleotide transhydrogenase, α-subunit), ΔpntB (pyridine nucleotide transhydrogenase, β-subunit), ΔudhA (soluble pyridine nucleotide transhydrogenase). (TIF) [file pone.0025573.s001.tif]

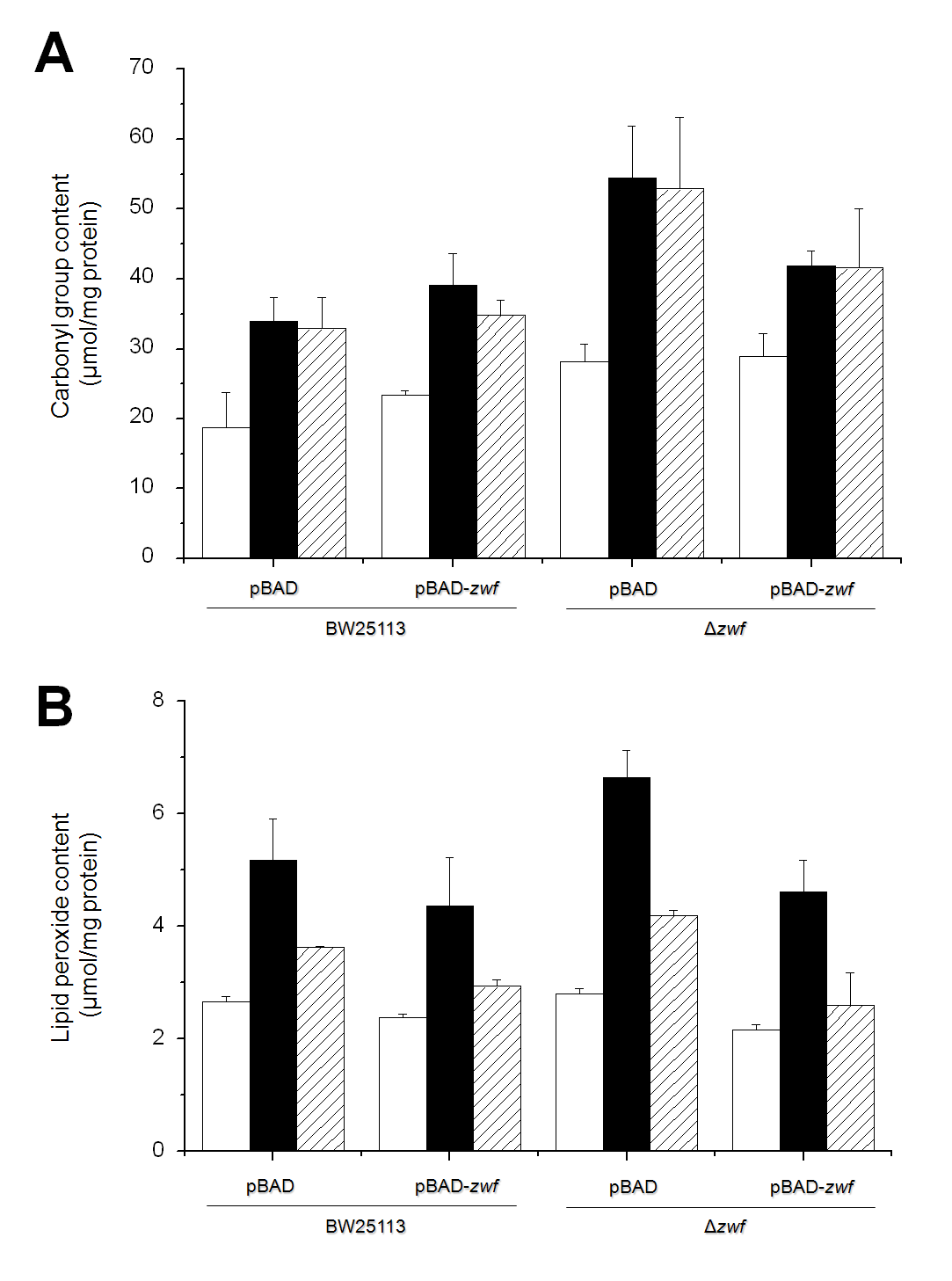

Supplement: Figure S2 — Effect of zwf expression on macromolecule oxidation. Oxidized cytoplasmatic proteins (A) and total membrane lipid peroxides (B) were assessed in the indicated strains. Cells were grown in LB-arabinose in the absence of toxicant (white bars) or exposed to 2 µM tellurite (black bars) or 100 µM H2O2 (stripes) for 30 min. Values are the average of 3 independent trials ± SD. (TIF) [file pone.0025573.s002.tif]

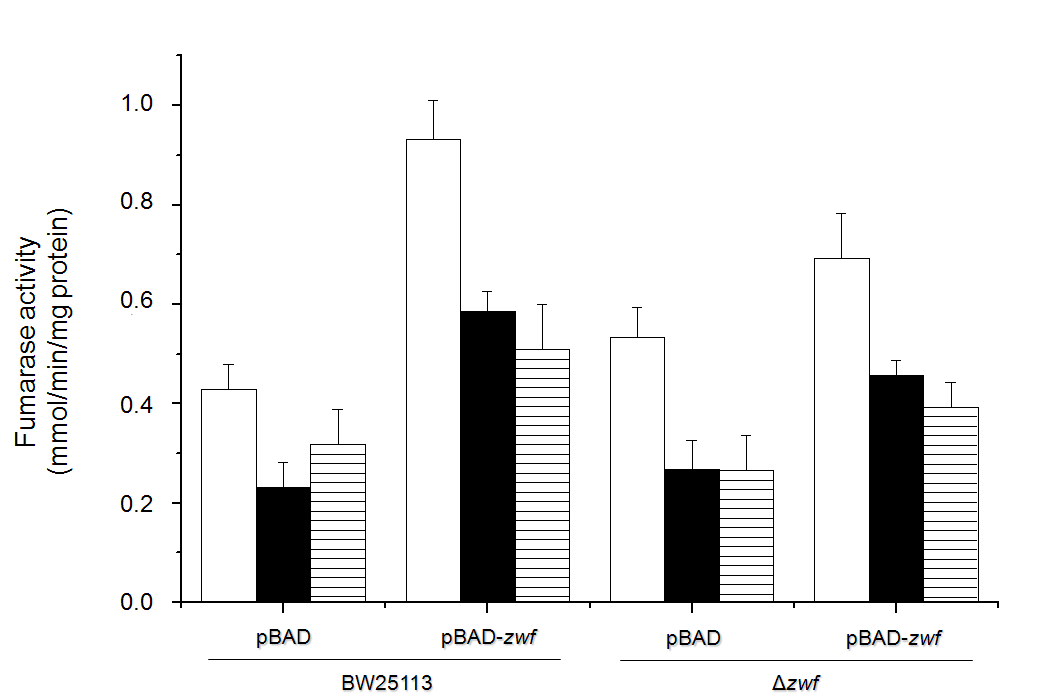

Supplement: Figure S3 — Effect of zwf expression on fumarase activity. Total fumarase activity was assessed as described in Methods. The indicated strains were grown in LB-arabinose in the absence of toxicants (white bars) or exposed for 30 min to 2 µM tellurite (black bars) or 100 µM menadione (stripes) for 30 min. Values are the average of 3 independent trials ± SD. (TIF) [file pone.0025573.s003.tif]
