# Supplementary material for: Glucose-6-Phosphate Dehydrogenase Protects Escherichia coli from Tellurite-Mediated Oxidative Stress
Source: PLoS One. 2011 Sep 30;6(9):e25573. doi: 10.1371/journal.pone.0025573 (PMC3184162; doi:10.1371/journal.pone.0025573)
Supplement: Table S1 — E. coli strains, plasmids and primers used in this study. (DOCX) [file pone.0025573.s004.docx]

Table S1. *E. coli* strains, plasmids and primers used in this study

| Strain | Relevant genotype | | Source or reference |
| --- | --- | --- | --- |
| TOP10 | F^-^ φ80*lac*ZΔM15 Δ*lac*Χ74 *rec*A1 *ara*D139 (Str^R^) *end*A1 | | Invitrogen® |
| BW25113 | ∆*(araD-araB)567*, ∆*lacZ4787*(::rrnB-3) | | [58] |
| pBAD | BW25113 harboring pBAD | | This work |
| pBAD-*zwf* | BW25113 harboring pBAD-*zwf* | | This work |
| Δ*zwf* | BW25113 *zwf* (*zwf*::Kan^R^) | | [58] |
| Δ*zwf* pBAD | BW25113 Δ*zwf* harboring pBAD | | This work |
| Δ*zwf* pBAD-*zwf* | BW25113 Δzwf harboring pBAD-*zwf* | | This work |
| Δ*gnd* | BW25113 *gnd* (*gnd*::Kan^R^) | | [58] |
| Δ*icdA* | BW25113 *icdA* (*icdA*::Kan^R^) | | [58] |
| Δ*maeB* | BW25113 *maeB* (maeB::Kan^R^) | | [58] |
| Δ*gdh* | BW25113 *gdh* (*gdh*::Kan^R^) | | [58] |
| Δ*pntA* | BW25113 *pntA* (*pntA*::Kan^R^) | | [58] |
| Δ*pntB* | BW25113 *pntB* (*pntB*::Kan^R^) | | [58] |
| Δ*sthA* | BW25113 *sthA* (*sthA*::Kan^R^) | | [58] |
| pET-G6PDH | BL21 (DE3) harboring pET-G6PDH-His_6_ plasmid | | This work |
| *zwf*::*lacZ* | GC4468 single chromosomal copy *zwf*::*lacZ* | | [38] |
| Plasmids | Plasmid features | | Source or reference |
| pBAD TOPO | Expression vector, ApR | | Invitrogen® |
| pBAD-*zwf* | pBAD harboring *E. coli* *zwf* gene | | This work |
| pET-G6PDH-His_6_ | pET-28b(+) harboring *E. coli* *zwf* gene | | [30] |
| Primers | Forward (F) or Reverse (R), to amplify | 5′-3′ Sequence | |
| *zwf* F | F, *zwf* | ATGGCGGTAACGCAAACA | |
| *zwf* R | R, *zwf* | CTCAAACTCATTCCAGCAACGA | |
| pBAD F | F, pBAD cloning | ATGCCATAGCATTTTTATCCATA | |
| pBAD R | R, pBAD cloning | GATTTTCAGCCTGATACAGATTAAAT | |

[58] Baba T, Ara T, Hasegawa M, Takai Y, Okumura Y, *et al*. (2006) Construction of *Escherichia coli* K-12 in-frame, single-gene knockout mutants: the Keio collection. *Mol Syst Biol* 2:2006.0008.
